# Gender Differences in the Allergic Response of Mice Neonatally Exposed to Environmental Tobacco Smoke

**DOI:** 10.1080/1044667021000003989

**Published:** 2002-03

**Authors:** Brian W. P. Seymour, Kathleen E. Friebertshauser, Janice L. Peake, Kent E. Pinkerton, Robert L. Coffman, Laurel J. Gershwin

**Affiliations:** 1 Department of Immunobiology DNAX Research Institute of Molecular and Cellular Biology 901 California Avenue Palo Alto CA 94304 USA; 2 Department of Pathology, Microbiology and Immunology University of California School of Veterinary Medicine One Shields Ave. Davis CA 95616 USA; 3 Department of Anatomy Physiology and Cell Biology University of California Davis CA 95616 USA; 4 Dynavax Technologies 717 Poller St. Suite 100 Berkeley CA 94710 USA

## Abstract

Exposure to environmental tobacco smoke (ETS) has been shown to increase allergic sensitization and reactivity and there has been some suggestion that the influence of ETS on the allergic response is dissimilar in males and females. It is to be determined whether gender differences exist in the IgE response to ovalbumin (OVA) sensitization following ETS exposure from the neonatal period through adulthood. To address this thesis, we examined gender differences in OVA sensitization of BALB/c mice housed from birth through adulthood under smoking and nonsmoking conditions. At 6 weeks of age (day 0) all mice were injected i.p. with OVA in aluminum hydroxide adjuvant followed by three 20 min exposures to 1% aerosolized OVA between day 14 and 80. There were significantly (*p*<0.05) more total and OVA specific IgE and IgG1 in the serum of females compared to males. Moreover, these sex responses, along with eosinophilia, were further enhanced in mice exposed to ETS. There were also significantly more IgE positive cells in the lungs of female, but not male, mice exposed to ETS compared with ambient air (*p*<0.05). There was also an elevation of Th2 cytokines (IL4, IL5, IL10, and IL13) after re-stimulation of lung homogenates following ETS exposure. These data demonstrate that female animals are significantly more susceptible than males to the influence of ETS on the allergic response.


*Developmental Immunology*, 2002 Vol. 9 (1), pp. 47–54

Taylor & Francis healthsciences

### Gender Differences in the Allergic Response of Mice Neonatally Exposed to Environmental Tobacco Smoke

BRIAN W.P. SEYMOUR[a,b], KATHLEEN E. FRIEBERTSHAUSER[b], JANICE L. PEAKE[c], KENT E. PINKERTON[c], ROBERT L. COFFMAN[d] and LAUREL J. GERSHWIN[b,*]

[a]*Department of Immunobiology, DNAX Research Institute of Molecular and Cellular Biology, 901 California Avenue, Palo Alto, CA 94304, USA;* [b]*Department of Pathology, Microbiology and Immunology, University of California, School of Veterinary Medicine, One Shields Ave., Davis, CA 95616, USA;* [c]*Department of Anatomy, Physiology and Cell Biology, University of California, Davis, CA 95616, USA;* [d]*Dynavax Technologies, 717 Poller St., Suite 100, Berkeley, CA 94710, USA*

Exposure to environmental tobacco smoke (ETS) has been shown to increase allergic sensitization and reactivity and there has been some suggestion that the influence of ETS on the allergic response is dissimilar in males and females. It is to be determined whether gender differences exist in the IgE response to ovalbumin (OVA) sensitization following ETS exposure from the neonatal period through adulthood. To address this thesis, we examined gender differences in OVA sensitization of BALB/c mice housed from birth through adulthood under smoking and nonsmoking conditions. At 6 weeks of age (day 0) all mice were injected i.p. with OVA in aluminum hydroxide adjuvant followed by three 20 min exposures to 1% aerosolized OVA between day 14 and 80. There were significantly ($p < 0.05$) more total and OVA specific IgE and IgG1 in the serum of females compared to males. Moreover, these sex responses, along with eosinophilia, were further enhanced in mice exposed to ETS. There were also significantly more IgE positive cells in the lungs of female, but not male, mice exposed to ETS compared with ambient air ($p < 0.05$). There was also an elevation of Th2 cytokines (IL4, IL5, IL10, and IL13) after re-stimulation of lung homogenates following ETS exposure. These data demonstrate that female animals are significantly more susceptible than males to the influence of ETS on the allergic response.

*Keywords*: Cytokines; Gender; *In vivo* animal models; Lung; Tobacco smoke; Th2

*Abbreviations*: AL, aluminum hydroxide; ETS, environmental tobacco smoke; NIP, nitroiodophenyl; OVA, ovalbumin; T, TWEEN 20

### INTRODUCTION

Several reports have shown that gender differences exist in the severity of many diseases involving the immune system due to sex-linked genetic and hormonal factors (Cua *et al.*, 1995; Beebe *et al.*, 1997; Bebo *et al.*, 1999; Ito *et al.*, 2001). This is particularly true for autoimmune diseases (Duquette *et al.*, 1992; Beeson, 1994). Furthermore, the clinical course of diseases such as multiple sclerosis have been shown to be influenced by hormones that can cause a shift in the balance of Th1/Th2 cytokines (reviewed in Ito *et al.*, 2001). In contrast, the role of gender as a factor in the development of allergic asthma and particularly in allergic enhancement by environmental tobacco smoke (ETS) exposure has not been well examined. Although, a genetic predisposition to the development of allergic asthma is recognized (Daniels *et al.*, 1996; Gern *et al.*, 1999) evidence strongly suggests that environmental agents such as ETS increase its phenotypic expression (el-Nawawy *et al.*, 1996; Gilliland *et al.*, 2001). Some studies have shown that males are more likely to be allergic from exposure to ETS or mainstream smoke than females (Ronchetti *et al.*, 1992; Omenaas *et al.*, 1994; Jarvis *et al.*, 1995), while others have reported no sex differences in the prevalence of allergy (Osaka *et al.*, 1985). These studies, while intriguing, are also controversial due to the multiple environmental factors that affect human beings, making it difficult to evaluate the true impact of ETS on the prevalence and severity of allergy.

We have previously shown that exposure of adult BALB/c mice to ETS enhances the allergic response in ovalbumin (OVA) sensitized mice when compared to those in ambient air (Seymour *et al.*, 1997). The ensuing response was characterized by enhancement of blood eosinophils, serum IgE, IgG1 and Th2 cytokines particularly IL4 and

*Corresponding author. Tel.: +1-530-752-6643. Fax: +1-530-752-3349. E-mail: ljgershwin@ucdavis.edu

ISSN 1044-6672 print © 2002 Taylor & Francis Ltd
DOI: 10.1080/1044667021000003989

IL10. In the present study, we have attempted to model the environment of a child born into a home of smokers and to compare this environment with a non-smoking one. To accomplish this, female BALB/c mice gave birth in either ETS or ambient air and their litters were raised in those environments. Mice of both sexes from these environments were sensitized to OVA and gender differences in the allergic response were examined. The allergic response was evaluated by quantification of the cytokines in the lungs, peripheral blood eosinophils, serum antibodies and immunohistochemistry with histological examination of the lungs.

### MATERIALS AND METHODS

### Animals

Pregnant pathogen-free BALB/c female mice were obtained from Animal Technolgies (Fremont, CA). They were housed in either ETS or ambient air and their litters were used in the experiment.

### Research Cigarettes

The cigarette used in this study was the IR4F, a filtered cigarette used for research produced by the Tobacco and Health Research Institute (University of Kentucky, Lexington, KY) and stored at 4°C until used. Two days before use, they were placed at 23°C in a chamber containing water and glycerol (at a ratio of 0.76:0.26) to achieve a relative humidity of 60%.

### ETS Exposure

The smoke generation system was designed by Teague *et al.* (1994). Whenever animals were not receiving ETS, filtered air was delivered to the mice. Mice were exposed to ETS for 6 h per day from Monday through Friday through adulthood but daily during the neonatal period between birth and 3 weeks of age, as shown in Fig. 1. At the end of the daily exposure, the smoke generator was turned off, but the animals remained in the exposure chambers. The total suspended particulates concentration in ETS was 1.01 ± 0.05 mg/in$^3$. The nicotine and carbon monoxide concentrations were 146 ± 54 μg/m$^3$ and 6.1 ± 0.7 ppm, respectively. The relative humidity was 52.5 ± 10.0%, while the temperature was 71.3 ± 1.5°F. Measurements of nicotine after the system was turned off revealed a rapid decline to nondetectable levels (<15 μg/in$^3$) during the nonsmoking period.

### Antigen Exposure and Immunization

At six weeks of age, the mice were injected i.p. with 10 μg OVA (Sigma, St. Louis, MO) in 2 mg of aluminum hydroxide adjuvant. The animals were later exposed to aerosolized OVA from 1% OVA (wt/vol.) in PBS (Fig. 1). Aerosolization was performed for 20 min using a Passport aerosol compressor (Invacare Corp, Elyria, OH) connected to a box 3 ft$^3$ in size which served as the deposition chamber for the mice.

### Immunohistochemical Evaluation of IgE Positive Cells in the Lung Parenchyma

On day 4 after the last aerosol, lungs from all groups were inflated and fixed with 1% paraformaldehyde at 30 cm water pressure for 1 h and placed into 70% ethanol until they were processed into paraffin. A rat anti-mouse antibody, EM95 (DNAX) was used to detect IgE positive cells in 5 μm thick paraffin sections. The sections were deparaffinized in xylene, rehydrated through a graded series of ethanol and treated with 3% hydrogen peroxide to block endogenous peroxidase activity. Nonspecific binding was blocked with 10% rabbit serum in 0.01 M PBS, pH 7.4, and sections were incubated in primary antibody diluted at 1:5000 in blocking serum for 1 h at 37°C. A biotinylated rabbit anti-rat, mouse adsorbed, secondary antibody was used, including Vectastain elite ABC immunoperoxidase reagents and a DAB peroxidase substrate kit (Vector Laboratories, Burlingame, CA). Hematoxylin was used as the nuclear counterstain. For a negative reagent control, the primary antibody was substituted with rat serum and used on OVA-challenged tissue sections. The negative control did not show positive staining for IgE positive cells.

To calculate the number of IgE positive cells present in lung parenchyma, 10 random, non-overlapping fields were examined. Fields containing large airways and blood vessels were excluded. Positive cells with abundant

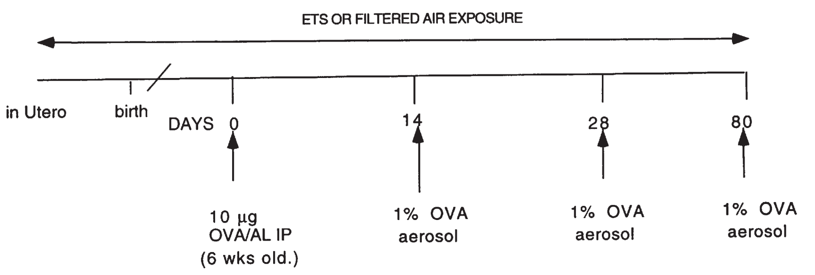

FIGURE 1 The time illustrates the exposure and sensitization protocol, beginning with pregnant females that were housed in either ETS or ambient air. Litters were retained in the same environment of the mother throughout the experiment.

amounts of cytoplasm strongly staining for IgE as well as positive cells with thin rims of cytoplasm strongly staining for IgE were observed and counted in the tissue sections. The final results were expressed as number of positive cells/10 fields.

### *In-vitro* Restimulation of Lung Cells

Mice were euthanized and their lungs removed aseptically, cut into small pieces and forced against a sterile No. 100 steel mesh (Tylinter Inc., Mentor, OH) with the piston of a 12 ml plastic syringe. The suspended cells were washed three times in Hanks solution and cells were stimulated in 1 ml cultures consisting of RPMI 1640 (J.H.R. Bioscience, Lenexa, KS) with 10% heat-inactivated FCS (Sigma, St. Louis, MO), 0.05 mM 2-ME (Sigma), 2 mM L-glutamine (J.H.R. Bioscience, Lenexa, KS) and penicillin/streptomycin (GIBCO BRL, Life Technologies Inc., Grand Island, NY). Lung cells were stimulated at $5 \times 10^6$/ml in culture medium containing 0.25 mg/ml OVA. The supernatant was harvested at 72 h.

### Cytokine Analysis

Sandwich ELISAs were performed to measure IL5, IL10 and IFN-γ as described (Abrams, 1995). IL-4 from cultured supernatant was detected by a bioassay using the IL4 dependent CT.4S cell line (kindly donated by Dr William Paul, NIH) as described (Seymour *et al.*, 1997).

### Analysis of Total IgE and OVA Specific Serum IgE

Total serum IgE was determined using a two-step sandwich ELISA as described (Coffman *et al.*, 1986). OVA-specific serum IgE was determined using a two-step sandwich ELISA (Seymour *et al.*, 1998). Briefly, the coating antibody was a monoclonal anti-IgE antibody called EM95. The serum samples were added and allowed to bind; subsequently the DIG-OVA was added. Final detection was accomplished with anti-digoxigenin-Fab fragments coupled to horseradish peroxidase.

### Analysis of OVA Specific IgG1

ELISA plates were coated with 5 μg/ml of OVA in PBS. The detecting antibody was a biotinylated rabbit anti-IgG1 (3099D) from DNAX which was used at 0.5 μg/ml. (Seymour *et al.*, 1998). The positive control for OVA specific IgG1 was pooled sera from hyperimmunized IgG1 standard run in parallel on anti-IgG1 coated plates.

### Quantification of OVA in the Lung of Aerosolized Mice

Immediately after a single OVA exposure, lungs were removed and a cell suspension was prepared for an OVA specific ELISA as previously described (Seymour *et al.*, 1998).

### Quantification of Eosinophils in the Peripheral Blood

Eosinophils were quantified by dilution of heparinized blood in Discombe's fluid (Colley, 1972).

### Statistics

Levels of antibodies, eosinophils and cytokines were calculated as mean and SEM. The two-tailed $p$ values were calculated according to the Mann–Whitney Test. Statistics for the morphometric analysis of IgE positive cells were performed using the Unpaired Students $t$-Test (Statview 4.5, Abacus Concepts, Inc. Berkeley, CA).

### RESULTS

### Gender Differences in the Antibody Levels from the Sera of OVA Sensitized Mice

By day 28, all mice exposed to ambient air produced increased levels of total and OVA specific IgE (Fig. 2).

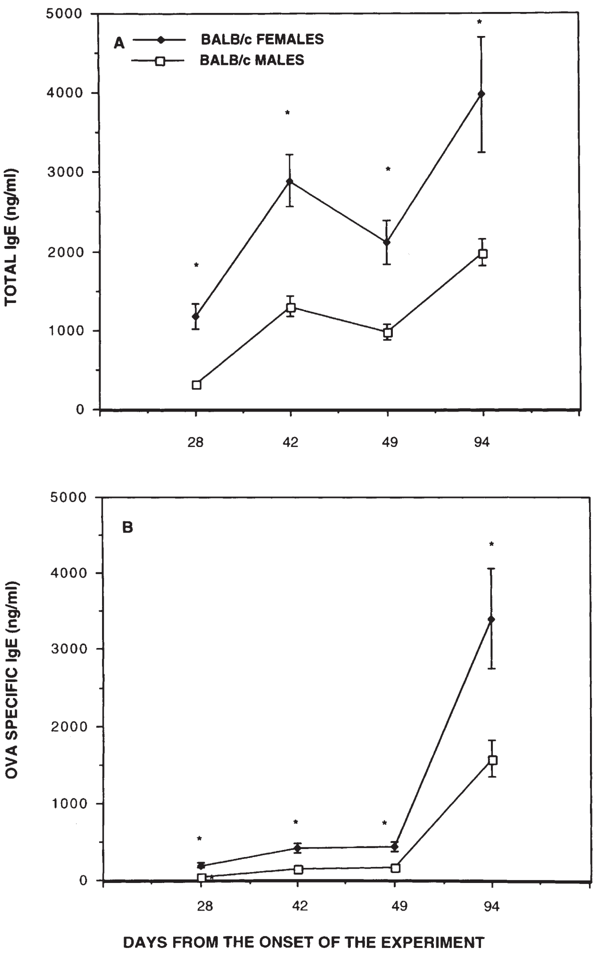

FIGURE 2 IgE levels of OVA immunized BALB/c mice exposed to ambient air. Mice were divided into two groups ($n = 20$). On day 0, all mice were immunized IP with OVA/AL followed by a 20 mm exposure to a 1% aerosolized OVA on days 14, 28 and 80. Mice were bled and the sera were tested for (A) total IgE in ng/ml. (B) OVA specific IgE ng/ml. Asterisks indicate $p < 0.05$.

However, at all time points, female mice made significantly more total and OVA specific IgE when compared to males ($p < 0.05$). The differences in OVA specific IgE between the sexes widened markedly at all time points assessed for IgE. For example, we observed approximately a four-fold increase in total and OVA specific IgE in female mice when compared to males on day 28. The largest increase in IgE was observed on day 94 (2 weeks after the final aerosolized OVA challenge). Here, there was approximately a two-fold increase in total and OVA specific IgE in female mice compared to males (total IgE in males was 1979 ± 170 ng/ml and OVA specific IgE was 1586 ± 240 ng/ml compared to 3980 ± 724 ng/ml and 3407 ± 656 ng/ml, respectively).

At the time of the last aerosol exposure (d80 from OVA/AL priming), all mice had elevated levels of OVA specific IgE (Fig. 3A). However, collectively, all mice that were housed in ETS had higher concentrations of OVA specific IgE than those in ambient air. Yet, the differences between ETS and ambient air groups were not statistically significant until the groups were divided by sex. Resulting comparisons then showed that the female mice in ETS made significantly more OVA specific IgE than all other groups including females housed in ambient air (OVA specific IgE concentrations in ETS females were 441 ± 142 ng/ml compared to 231 ± 67 ng/ml from females in ambient air, $p = 0.03$). Maximal responses with respect to OVA specific IgE were observed seven days after the last aerosolized OVA exposure (Fig. 3A). Female mice housed in ETS had significantly higher serum concentrations of OVA specific IgE than all male mice. However, at this time point, levels of significance were not attained when females in ETS were compared to females in ambient air. Although males housed in ETS also had higher serum concentrations of OVA specific IgE than males in ambient air, these data did not attain levels of significance (IgE concentrations in ETS exposed males were 1465 ± 295 ng/ml compared to 852 ± 191 ng/ml, $p = 0.11$, respectively). Female mice in ambient air had much greater serum concentrations of OVA specific IgE than males in ambient air (1976 ± 283 ng/ml vs. 852 ± 191 ng/ml, $p = 0.0008$, respectively). However, when female mice in ambient air were compared with males in ETS at the same time point, there was no longer a significant difference in OVA specific IgE between them (1976 ± 283 ng/ml vs. 1465 ± 295 ng/ml, respectively, $p = 0.222$) due to the enhancing effect of ETS on the IgE response. Moreover, females in ETS had significantly higher serum concentrations of OVA specific IgE that males housed in the same environment (3296 ± 555 vs. 1465 ± 295, respectively, $p = 0.019$).

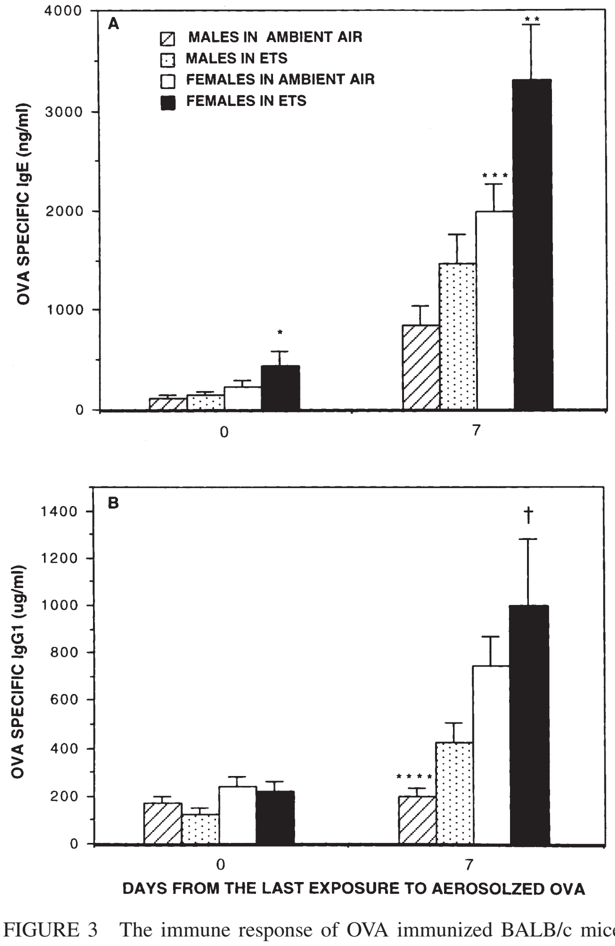

FIGURE 3 The immune response of OVA immunized BALB/c mice after long-term exposure to ETS or ambient air. (A) OVA specific IgE (ng/ml). (B) OVA specific IgG1 μg/ml. $^{*}p = 0.03$ vs. females in ambient air. $^{**}p = 0.019$ vs. males in ETS. $^{***}p = 0.0008$ vs. males in ambient air. $^{****}p = 0.002$ vs. males in ETS. $^{\dagger}p < 0.05$ compared to males in ETS or ambient air.

Significant gender differences in OVA specific IgG1 were observed only on day 7 after the last exposure to aerosolized OVA (Fig. 3B). At this time, females housed in ETS showed the highest response when compared to all other groups. The OVA specific serum IgG1 concentrations in females were significantly higher than those in males regardless of ETS or ambient air exposure ($p < 0.005$). However, among mice of the same sex, significant differences relevant to ETS or ambient air exposure were seen only between the males. Thus, male mice housed in ETS had a mean serum concentration of 427 ± 80 μg/ml OVA specific IgG1, while males in ambient air had a mean serum concentration of 197 ± 37 μg/ml OVA specific IgG1 ($p = 0.002$).

### IgE Positive Cells in the Lung Parenchyma of OVA Sensitized Mice Exposed to ETS or Ambient Air

Mononuclear cells that stained positive for IgE were found throughout the lung parenchyma, in the perivascular influx of inflammatory cells and occasionally in the airway epithelium and lamina propria of OVA sensitized mice. Morphometric analysis of the lung parenchyma demonstrated that exposure to ETS significantly increased ($p < 0.05$) the number of IgE positive cells in females sensitized with OVA compared to females that were housed in ambient air (Fig. 4). There was no statistically significant

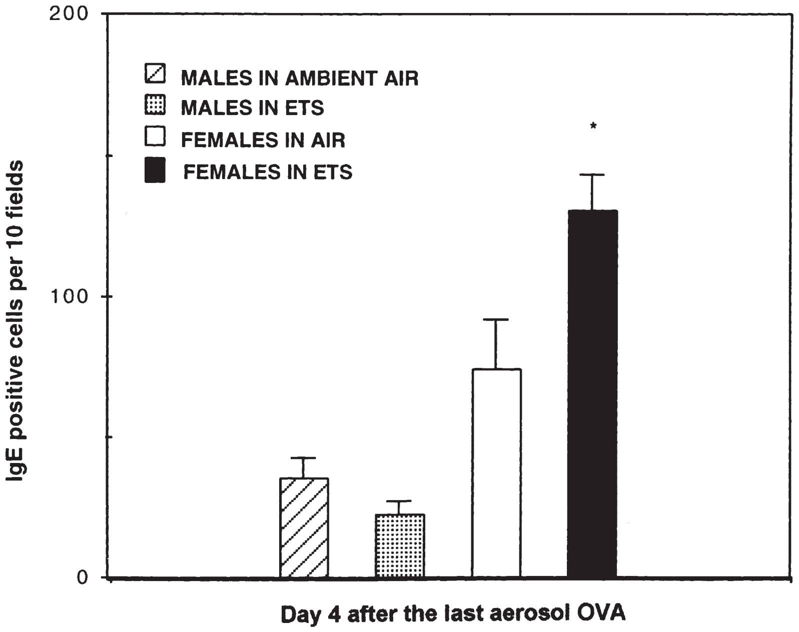

FIGURE 4 IgE positive cells in the lung parenchyma of OVA sensitized mice exposed to ETS/ambient air. Four days after the last aerosol OVA, lungs were removed and tested for IgE positive cells. Asterisks indicate $p < 0.05$.

difference in the number of IgE positive cells between OVA sensitized males exposed to ETS or ambient air. Mice not sensitized with OVA had few IgE positive cells (not shown).

### Eosinophils in the Peripheral Blood

Quantitative differences in eosinophilia were not observed between the genders at the early time points. Thus, results from both sexes were pooled and mice in ETS were compared with those in ambient air (Fig. 5). Mice (naïve with respect to OVA) exposed to ETS, from birth to adulthood at 6 weeks of age, showed a significant enhancement of eosinophils in the blood when compared to similar naïve mice in ambient air ($53 \pm 5 \times 10^{-4}$/ml vs. $27 \times 4 \times 10^{-4}$/ml, respectively, $p = 0.0002$). Exposure of mice to OVA led to additional enhancement of blood eosinophils from ETS housed mice as seen at day 21 and again at day 36. At day 36, extremely significant differences in eosinophils were observed when ETS exposed mice were compared to mice housed in ambient air ($184 \pm 14 \times 10^{-4}$/ml vs. $107 \pm 13 \times 10^{-4}$/ml, $p = 0.0006$). By this time, significant differences were apparent in blood eosinophil levels between genders; this was particularly evident in the data obtained from the males and females housed in ambient air. Female mice had

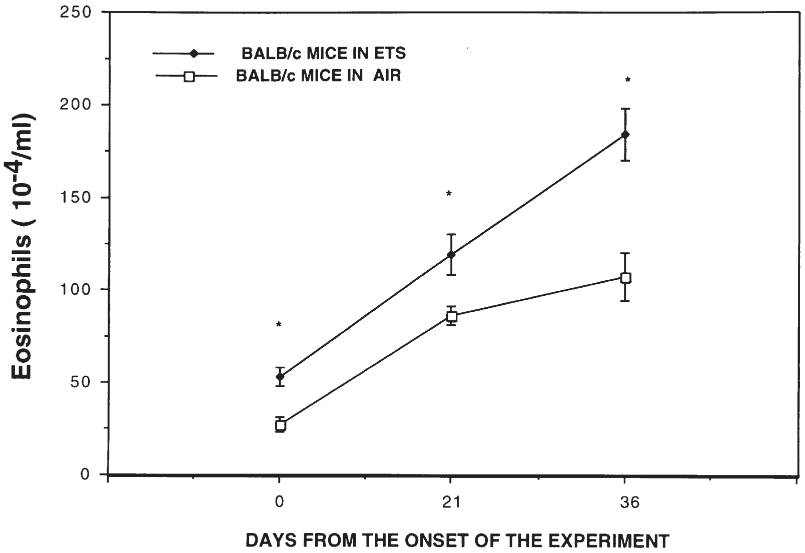

FIGURE 5 Eosinophil levels in the blood of OVA/AL sensitized mice housed in either ETS or ambient air since birth after exposure to aerosolized OVA. Asterisks indicate $p < 0.05$.

TABLE I OVA specific cytokines in the lung of OVA sensitized mice exposed to ETS/ambient air

| Gender | Exposure | IL4 (pg/ml) | IL13 (pg/ml) | IL10 (U/ml) | IL5 (pg/ml) | IFN-$\gamma$ (pg/ml) |
|---|---|---|---|---|---|---|
| Male (9) | Air | 140 ± 66 | 855 ± 23 | 0.943 ± 0.21 | 404 ± 13 | $<156$ |
| Male (12) | ETS | 252 ± 43* | 1499 ± 263* | 1.420 ± 0.19* | 1003 ± 100* | $<156$ |
| Female (9) | Air | 187 ± 37 | 2891 ± 324 | 2.470 ± 0.26 | 3099 ± 309 | $<156$ |
| Female (9) | ETS | 404 ± 63** | 3079 ± 252 | 3.594 ± 0.65 | 3328 ± 487 | $<156$ |

*In vitro* stimulation to detect lung cytokines was done on day 7 after the last aerosolized OVA exposure.
*$p < 0.05$ compared to newborn males in air.
**$p < 0.05$ when compared to newborn females in air.
Data are presented as means ± standard error.

a mean eosinophil level of $157 \pm 13 \times 10^{-4}$/ml, while male mice had a mean level of $60 \pm 8 \times 10^{-4}$/ml eosinophils ($p < 0.0001$).

Eosinophils were found throughout the lung parenchyma of the OVA sensitized mice. The groups that were not sensitized with OVA did not show evidence of lung inflammation and eosinophils were rarely seen (not shown).

### Gender Differences in Lung Levels of Cytokines

On day 87, lungs were removed from all mice and cell suspensions of the whole lungs and associated lymph nodes were restimulated *in vitro* for 72 h with OVA; supernatants of these cultures were assayed for cytokines (Table I). A polarized Th2 response with no detectable IFN-$\gamma$ was apparent in all mice. Greater concentrations of cytokines were detected from cells of female than from those of males from the same environment. Although the IL-4 concentration produced by cells from ambient air housed females was greater than that from similarly housed males, the difference was not significant. However, when concentrations of IL13 were measured extremely significant ($p < 0.0001$) differences were observed. Moreover, extremely significant differences were also observed between males and females housed in ambient air for IL5 ($p < 0.0001$) and IL10 ($p = 0.0008$).

The enhancing effect of ETS on the cytokine concentrations was most apparent for males. All cytokines examined, except for IFN-$\gamma$, were significantly enhanced in ETS exposed males when compared to males in ambient air ($p < 0.05$). However, when the effect of ETS on cytokine production in females was examined, the greatest enhancements were seen in IL4 and IL10, when ETS housed females were compared to females in ambient air (Table I).

### Deposition of OVA in Lung

The quantity of OVA that was deposited in the lung of mice from both sexes was determined after 20 min of exposure to a 1% aerosolized OVA (Fig. 6A). Six week old male mice received 2588 ± 300 ng of OVA, while female mice received 2120 ± 200 μg OVA. A greater quantity of OVA was deposited in the lung of 12 weeks old mice of both sexes when compared to 6 weeks old mice. Although male mice consistently inhaled greater quantities of OVA than females, these differences were not significant. The clearance of OVA from the lung of 6 weeks old females was rapid. At 48 h after the mice inhaled 2000 ng of OVA, 99.5% of the antigen was cleared from the lung (Fig. 6B). The quantity of OVA remaining in the lung at 72 h was 6.3 ± 0.9 ng. Results from males exposed to a 1% aerosolized OVA for 20 min revealed the same clearance

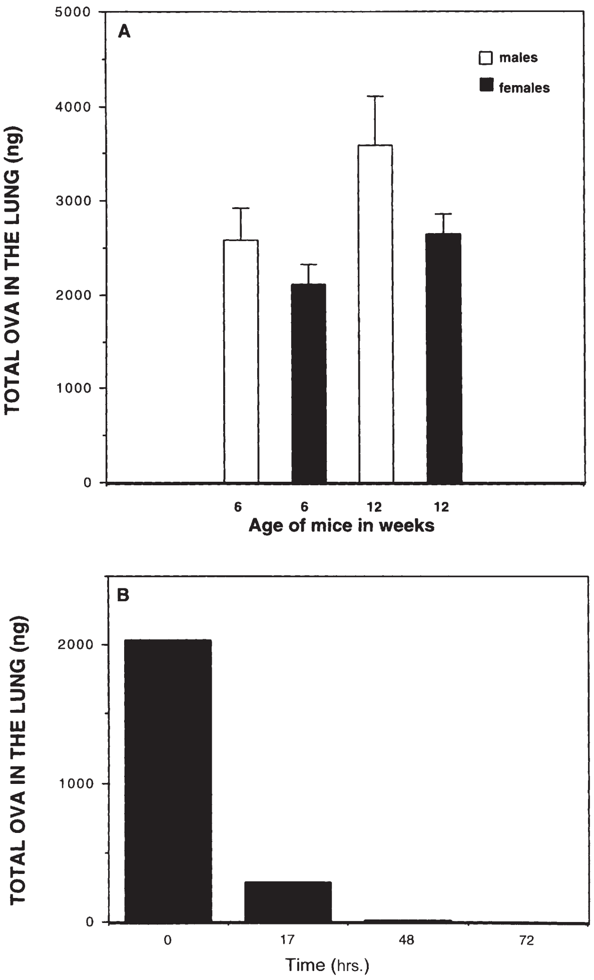

FIGURE 6 Measurements of OVA in the lung of BALB/c mice after exposure to a 1% aerosolized OVA. (A) Clearance of OVA from the lung of BALB/c mice after receiving 2000 ng of OVA. (B) Mice were exposed to OVA aerosol and lungs were removed immediately, minced, and then suspended in 10 ml. of RPMI. Suspension was centrifuged and supernatant assayed by ELISA for OVA. Ten mice of each gender were used in part A and an average of two mice/time point were sacrificed in part B, except for the 74 h time point in which five mice/group were used.

rate as seen in females; 72 h after exposure, there were 4.4 ± 1.0 ng of OVA in the lungs.

### DISCUSSION

Mice born into and raised in ETS are a unique model for the human infant and child born into and raised in a home of smokers. Using this model we tested our earlier hypothesis that male and female children will respond differently to the Th2 adjuvant effect of ETS (Seymour *et al.*, 1997). The data demonstrate a significant difference in OVA sensitization of females when compared to males in ambient air. This difference is based on Th2 cytokines and IgE and IgG1 responses to OVA sensitization. This enhancement effect in females was seen with multiple parameters of the allergic response including: IgE, IgG1, Th2 cytokines, peripheral blood eosinophil counts, and cells staining for IgE within the lung. The combination of female gender and ETS exposure resulted in the highest IgE concentrations and the greatest number of IgE containing cells in the lung. However, the effect of ETS exposure on the males was significant for all of the Th2 cytokines, indicating that even through the levels of cytokines produced were lower in males than in females, the ETS exposure effectively increased those levels.

These observations raise questions concerning the mechanisms involved in this gender difference. One potential mechanism we considered was that differences in lung size, development, or breathing pattern could cause males and females to inhale different amounts of aerosolized OVA resulting in different amounts being deposited in the lung. Data obtained to test this hypothesis was not supportive of this mechanism. Hormones may also be partly responsible for the modulation of the allergic response and several hormones have been implicated in enhancing the activity of Th cells (discussed in Romagnani, 1994). *In vitro*, progesterone has been shown to cause human Th cells to produce Th2 cytokines (Hamano *et al.*, 1998). Other investigators have speculated that the maternal immune response is biased towards humoral immunity and away from cell mediated immune responses; cytokines such as IFN-$\gamma$ have been shown to be deleterious in the microenvironment during pregnancy (Chaouat *et al.*, 1990). Indeed, investigators have also shown that Th2 cytokines are up regulated in female mice during pregnancy (Wegmann *et al.*, 1993).

Patients with multiple sclerosis and other cell mediated autoimmune diseases involving Th1 lymphocytes (Steinman, 1996) go into remission during pregnancy probably due to increases in the production of sex hormones which down regulate Th1 responses (Ito *et al.*, 2001). In a murine model of experimental autoimmune encephalomyelitis, induced by myelin basic protein, female mice are more susceptible than males. Furthermore, a reduction in the severity of disease occurs in females implanted with dihydrotestosterone pellets (Dalal *et al.*, 1997). Others have shown that this protection is lost in castrated males, thereby demonstrating gender-related differences due to hormonal influences in disease processes (Bebo *et al.*, 1999). Herein, we demonstrate that even in the absence of ETS, the Th2 cytokine response and the allergic phenotype appears to be greater in the female when compared to males. In particular, IL5 and IL13 concentrations were approximately eight and three-fold higher in females than males, respectively. Blood eosinophilia has been shown to be a risk factor in acute asthma (Janson and Herala, 1992); IL13 is a key cytokine in the pathogenesis of allergic asthma (Grunig *et al.*, 1998).

In a report by the American Thoracic Society, deaths from asthma between 1979 and 1992 were significantly greater among females than males (Society, 1995). Therefore, it is possible that the Th2 bias of females, in conjunction with environmental factors such as ETS and diesel exhaust particles that can enhance the Th2 response (Diaz-Sanchez *et al.*, 1997), may play a role in the increased mortality from allergic asthma as seen in females. An epidemiological study on the relationship of active and passive exposure to tobacco smoke on total IgE and asthma in human subjects further supports the validity of this murine model. Specifically, it was found that while both men and women, who were first-degree relatives of asthmatics had increased total serum IgE as a result of passive smoke exposure, the relationship was only statistically significant in the women (Oryszczyn *et al.*, 2000). In summary, the murine data presented herein provides support for the hypothesis that early and sustained exposure to ETS stimulates development of an allergic phenotype and that there is a gender bias for this effect.

### Acknowledgements

The authors thank Michael Goldsmith and Steve Teague for their technical assistance with the cigarette smoke generator system and the excellent care of the animals while they were housed in the exposure chambers. Animals were cared for under NIH guidelines. Animal care protocol was approved by the University of California, Davis Committee on Animal Use and Care. We thank Drs Amy Beebe, Dan Cua and Patricia Gilford for constructive comments on the manuscript.

This research was supported by the University of California, Tobacco Related Disease Research Program.

### References

Abrams, J. (1995) In: Roico, P., ed, *Current Protocols in Immunology* (John Wiley and Sons, Inc., New York) **6**, p 6:20:1.

Bebo, Jr, B.F., Adlard, K., Schuster, J.C., Unsicker, L., Vandenbark, A.A. and Offner, H. (1999) “Gender differences in protection from EAE induced by oral tolerance with a peptide analogue of MBP-Ac1-11”, *J. Neurosci. Res.* **55**, 432–440.

Beebe, A.M., Mauze, S., Schork, N.J. and Coffman, R.L. (1997) “Serial backcross mapping of multiple loci associated with resistance to *Leishmania major* in mice”, *Immunity* **6**, 551–557.

Beeson, P.B. (1994) “Age and sex associations of 40 autoimmune diseases”, *Am. J. Med.* **96**, 457–462.

Chaouat, G., Menu, E., Clark, D.A., Dy, M., Minkowski, M. and Wegmann, T.G. (1990) "Control of fetal survival in CBA $\times$ DBA/2 mice by lymphokine therapy", *J. Reprod. Fertil.* **89**, 447–458.

Coffman, R.L. and Carty, J. (1986) "A T cell activity that enhances polyclonal IgE production and its inhibition by interferon-gamma", *J. Immunol.* **136**, 949–954.

Colley, D.G. (1972) "Schistosoma mansoni: eosinophilia and the development of lymphocyte blastogenesis in response to soluble egg antigen in inbred mice", *Exp. Parasitol.* **32**, 520–526.

Cua, D.J., Hinton, D.R., Kirkman, L. and Stohlman, S.A. (1995) "Macrophages regulate induction of delayed-type hypersensitivity and experimental allergic encephalomyelitis in SJL mice", *Eur. J. Immunol.* **25**, 2318–2324.

Dalal, M., Kim, S. and Voskuhl, R.R. (1997) "Testosterone therapy ameliorates experimental autoimmune encephalomyelitis and induces a T helper 2 bias in the autoantigen-specific T lymphocyte response", *J. Immunol.* **159**, 3–6.

Daniels, S.E., Bhattacharrya, S., James, A., Leaves, N.I., Young, A., Hill, M.R., Faux, J.A., Ryan, G.F., le Souef, P.N., Lathrop, G.M., Musk, A.W. and Cookson, W.O. (1996) "A genome-wide search for quantitative trait loci underlying asthma", *Nature* **383**, 247–250.

Diaz-Sanchez, D., Tsien, A., Fleming, J. and Saxon, A. (1997) "Combined diesel exhaust particulate and ragweed allergen challenge markedly enhances human *in vivo* nasal ragweed-specific IgE and skews cytokine production to a T helper cell 2-type pattern", *J. Immunol.* **158**, 2406–2413.

Duquette, P., Pleines, J., Girard, M., Charest, L., Senecal-Quevillon, M. and Masse, C. (1992) "The increased susceptibility of women to multiple sclerosis", *Can. J. Neurol. Sci.* **19**, 466–471.

Gern, J.E., Lemanske, Jr, R.F. and Busse, W.W. (1999) "Early life origins of asthma", *J. Clin. Investig.* **104**, 837–843.

Gilliland, F.D., Li, Y.F. and Peters, J.M. (2001) "Effects of maternal smoking during pregnancy and environmental tobacco smoke on asthma and wheezing in children", *Am. J. Respir. Crit. Care Med.* **163**, 429–436.

Grunig, G., Warnock, M., Wakil, A.E., Venkayya, R., Brombacher, F., Rennick, D.M., Sheppard, D., Mohrs, M., Donaldson, D.D., Locksley, R.M. and Corry, D.B. (1998) "Requirement for IL-13 independently of IL-4 in experimental asthma", *Science* **282**, 2261–2263.

Hamano, N., Terada, N., Maesako, K., Hohki, G., Ito, T., Yamashita, T. and Konno, A. (1998) "Effect of female hormones on the production of IL-4 and IL-13 from peripheral blood mononuclear cells", *Acta. Oto-laryngol. Suppl.* **537**, 27–31.

Ito, A., Bebo, Jr, B.F., Matejuk, A., Zamora, A., Silverman, M., Fyfe-Johnson, A. and Offner, H. (2001) "Estrogen treatment down-regulates TNF-alpha production and reduces the severity of experimental autoimmune encephalomyelitis in cytokine knockout mice", *J. Immunol.* **167**, 542–552.

Janson, C. and Herala, M. (1992) "Blood eosinophil count as risk factor for relapse in acute asthma", *Respir. Med.* **86**, 101–104.

Jarvis, D., Luczynska, C., Chinn, S. and Burney, P. (1995) "The association of age, gender and smoking with total IgE and specific IgE", *Clin. Exp. Allergy* **25**, 1083–1091.

el-Nawawy, A., Soliman, A.T., el-Azzouni, O., Amer el, S., Demian, S. and el-Sayed, M. (1996) "Effect of passive smoking on frequency of respiratory illnesses and serum immunoglobulin-E (IgE) and interleukin-4 (IL-4) concentrations in exposed children", *J. Trop. Pediatr.* **42**, 166–169.

Omenaas, E., Bakke, P., Elsayed, S., Hanoa, R. and Gulsvik, A. (1994) "Total and specific serum IgE levels in adults: relationship to sex, age and environmental factors", *Clin. Exp. Allergy* **24**, 530–539.

Oryszczyn, M.P., Annesi-Maesano, I., Charpin, D., Paty, E., Maccario, J. and Kauffmann, F. (2000) "Relationships of active and passive smoking to total IgE in adults of the epidemiological study of the genetics and environment of asthma, bronchial hyperresponsiveness, and atopy (EGEA)", *Am. J. Respir. Crit. Care Med.* **161**, 1241–1246.

Osaka, F., Kasuga, H., Sugita, M., Matsuki, H. and Miyake, T. (1985) "A study of the relationship between mite IgE in the serum of school children and smoking habits in mothers", *Nippon Eiseigaku Zasshi* **40**, 789–795.

Romagnani, S. (1994) "Regulation of the development of type 2 T-helper cells in allergy", *Curr. Opin. Immunol.* **6**, 838–846.

Ronchetti, R., Bonci, E., Cutrera, R., De Castro, G., Indinnimeo, L., Midulla, F., Tancredi, G. and Martinez, F.D. (1992) "Enhanced allergic sensitisation related to parental smoking", *Arch. Dis. Child* **67**, 496–500.

Seymour, B.W., Pinkerton, K.E., Friebertshauser, K.E., Coffman, R.L. and Gershwin, L.J. (1997) "Second-hand smoke is an adjuvant for T helper-2 responses in a murine model of allergy", *J. Immunol.* **159**, 6169–6175.

Seymour, B.W., Gershwin, L.J. and Coffman, R.L. (1998) "Aerosol-induced immunoglobulin (Ig)-E unresponsiveness to ovalbumin does not require CD8+ or T cell receptor (TCR)-gamma/delta + T cells or interferon (IFN)-gamma in a murine model of allergen sensitization", *J. Exp. Med.* **187**, 721–731.

Society, A.T. (1995) "Future directions for research on diseases of the lung", *Am. J. Respir. Crit. Care Med.* **152**, 1713.

Steinman, L. (1996) "Multiple sclerosis: a coordinated immunological attack against myelin in the central nervous system", *Cell* **85**, 299–302.

Teague, S.V., Pinkerton, K.E., Goldsmith, M., Gebremichael, A., Chang, S., Jenkins, P.A. and Moneyhun, J.H. (1994) "A sidestream cigarette smoke generation and exposure system for environmental tobacco smoke studies", *Inhalation Toxicol.* **6**, 79.

Wegmann, T.G., Lin, H., Guilbert, L. and Mosmann, T.R. (1993) "Bidirectional cytokine interactions in the maternal-fetal relationship: is successful pregnancy a TH2 phenomenon?", *Immunol. Today* **14**, 353–356.

